# *Smo* gene silencing: a promising strategy for natural killer/t-cell lymphoma treatment via modulating proliferation and apoptosis

**DOI:** 10.1186/s10020-025-01341-z

**Published:** 2025-10-29

**Authors:** Shengquan Liu, Shaoxiong Wang, Yahong Xu, Yuanling Huang, Pengliang Xin, Yan Zheng, Yishen Wu, Yanling Yang, Xiongpeng Zhu, Chuntuan Li

**Affiliations:** Department of Hematology, First Hospital of Quanzhou Affiliated to Fujian Medical University, 248 East Street, Licheng District, Quanzhou, 362000 Fujian Province China

**Keywords:** *Smo* silencing, Natural Killer/T-Cell lymphoma, Cell proliferation, Cell apoptosis, Gene therapy, Xenotransplantation

## Abstract

Natural killer/T-cell lymphoma (NKTCL) is a malignancy with a poor prognosis. The Smoothened (Smo) protein is implicated in NKTCL growth. This study employed lentiviral vector-mediated *Smo* RNA interference (LV-*Smo*-RNAi) to silence the *Smo* gene in the human NKTCL cell line SNT8. Fluorescence microscopy, qRT-PCR, and Western blot verified the reduction of *Smo* mRNA and protein levels. The CCK-8 assay showed that *Smo* silencing inhibited cell proliferation. Flow cytometry with annexin V-PE/7-AAD double staining indicated an increased apoptosis rate. Moreover, the expression of GLI family zinc finger 1 (Gli1) and programmed death-ligand 1 (PD-L1) was downregulated. In vivo, xenotransplantation experiments demonstrated that *Smo* silencing led to slower tumor growth with reduced tumor volume and weight. Overall, *Smo* gene silencing holds great potential as a novel molecular-targeted therapy approach for NKTCL by effectively suppressing cell proliferation and promoting apoptosis.

**Supplementary information:**

The online version contains supplementary material available at 10.1186/s10020-025-01341-z.

## Highlights

1. This study is the first to report the anti-tumor effects of *Smo* gene silencing in the human NKTCL cell line SNT8

2. This research finds that *Smo* gene silencing significantly suppresses cell proliferation.

3. This research observes that *Smo* silencing significantly increases the apoptosis rate of NKTCL cells.

4. *Smo* silencing downregulates the expression of Gli1 and PD-L1, providing new insights into the underlying mechanisms.

5.This study provides a new molecular target for developing targeted therapies for NKTCL.

**Supplementary information:**

The online version contains supplementary material available at 10.1186/s10020-025-01341-z.

## Introduction

Natural killer T-cell lymphoma (NKTCL) is a rare but highly aggressive subtype of non-Hodgkin lymphoma, predominantly affecting patients in Asia and Latin America (Yan 2023). Its pathogenesis is complex (Cortés 2020), and resistance to conventional radiotherapy and chemotherapy contributes to its poor prognosis (Küçük et al., 2020. In recent years, advances in molecular targeted therapies have drawn increasing attention to the role of tumor-associated signaling pathways in NKTCL progression (Somasundaram et al.,^2019^.

The Hedgehog signaling pathway, a highly conserved developmental pathway, plays a critical role in the tumorigenesis of various cancers (Chen et al. 2023). Smoothened (Smo), a key transmembrane protein in this pathway, activates downstream Gli1 transcription factors, promoting their nuclear translocation and inducing the expression of proliferation-associated genes such as Cyclin D1 and Ki67 (Lin 2017; Qu 2021). Smo also upregulates anti-apoptotic proteins like Bcl-2 via Gli-dependent mechanisms, inhibiting apoptosis (Wu et al. 2023; Xu et al. 2016). Additionally, Smo antagonists have been shown to promote lipid accumulation by downregulating Gli1, suggesting that Smo activity may negatively regulate cellular differentiation (Qu 2021) Somasundaram et al.,^2019^. However, the specific role of the Hedgehog pathway in NKTCL remains poorly understood, particularly the function of Smo in regulating cell proliferation and apoptosis (Liu and (Liu 2018). Investigating the biological role of Smo may offer novel insights for molecular-targeted therapies in NKTCL.

Existing literature indicates that the Hedgehog signaling pathway is critical in various malignant tumors, particularly in solid tumors such as pancreatic cancer, lung cancer, and glioblastoma (Yang 2020). Studies have shown that excessive activation of the Smo protein can lead to abnormal proliferation of tumor cells and inhibit cell apoptosis (Wang 2023). This phenomenon has prompted the development of various Smo inhibitors, which have shown promising anti-tumor activity in several clinical trials (Martelli et al. 2023). However, research on the function of Smo protein in NKTCL is very limited (Wang 2023). Although some preliminary studies suggest that the Hedgehog signaling pathway may play a role in the occurrence and development of NKTCL (Liu and (Liu 2018), there is a lack of specific research on the Smo gene and its downstream signaling molecules. Additionally, existing treatment strategies for NKTCL have been unsatisfactory, highlighting the urgent need to develop new molecular targets (Sari et al. 2018). Therefore, exploring the function of the Smo gene in NKTCL cells holds significant theoretical and clinical relevance.

With the rapid development of genetic technology, RNA interference (RNAi) has become a powerful tool for studying the function of specific genes (Sumimoto and Kawakami 2007). In recent years, lentivirus-mediated RNAi technology has been widely used in tumor gene function research. Through the lentiviral vector-mediated *Smo* RNA interference (LV-*Smo*-RNAi) lentiviral vector, effective silencing of the *Smo* gene in NKTCL cells can be achieved, thereby inhibiting the expression of the Smo protein and allowing observation of its effects on cell proliferation, apoptosis, and tumor formation. Unlike traditional drug inhibitors, RNAi technology has high specificity and efficiency, enabling the specific silencing of target genes while reducing the impact on non-target genes. However, the application of RNAi technology in the field of NKTCL is still in its early stages, and there are no systematic studies investigating the effects of Smo gene silencing on the biological characteristics of NKTCL (Liu et al.^2019^. Therefore, using this technology to study the role of the *Smo* gene in NKTCL cells holds significant innovative and exploratory value.

To address this knowledge gap, we aimed to investigate the specific role of *Smo* gene silencing in regulating NKTCL cell proliferation and apoptosis and to elucidate its potential involvement in NKTCL tumorigenesis. This study not only contributes to a deeper understanding of the Hedgehog signaling pathway in cancer but also identifies Smo as a promising therapeutic target. Our findings provide a scientific rationale for Smo-targeted interventions in NKTCL, which may offer novel treatment strategies for patients with limited therapeutic options and poor prognosis.

## Materials and methods

### Culture and treatment of NKTCL cell lines

Human NKTCL cell lines SNT8 (provided by Xiamen Lianyuan Technology Co., Ltd.) and KHYG-1 (JCRB cell bank, JCRB0156) were cultured in RPMI-1640 medium (Thermo Fisher Scientific, 11875093, USA). The medium was supplemented with 10% fetal bovine serum (Gibco, 16000044, USA). Cells were maintained at 37 °C in a humidified incubator with 5% CO_2_. Jurkat T-cells (ATCC, TIB-152, USA) were cultured in RPMI-1640 medium (Therm Fisher Scientific, 11875093, USA) supplemented with 10% FBS (Gibco, 16000044, USA) and 1% penicillin-streptomycin (Therm Fisher Scientific, 15140122, USA) under the same conditions of 37 °C and 5% CO_2_. The culture medium was replaced every 2 to 3 days, and all SNT8 cells used in the experiments were in the logarithmic growth phase.

Cell Grouping: The groups included untreated SNT8/KHYG-1 cells, SNT8/KHYG-1 cells carrying shControl, and SNT8/KHYG-1 cells carrying shSmo. Drug treatment: The Smo agonist SAG (566661, Merck, USA) was applied to the shSmo group, while the Smo inhibitor Cyclopamine (239803, Merck, USA) was used in the shControl group. For dose-response experiments, cells were treated with SAG at 200, 300, 400, 500, 700, 800, and 1000 nM or with Cyclopamine at 0.1, 1, 2, 5, 7.5, and 10 µM for 24 h. NKTCL Treatment Drugs: The groups were treated with Doxorubicin (D1515, Sigma-Aldrich, USA) and Etoposide (E1383, Sigma-Aldrich, USA). Doxorubicin was administered at different concentration gradients: 0.1, 0.5, and 1 µM; Etoposide at 1, 5, and 10 µM, all for 24 h of treatment.

Co-culture: The human NKTCL cell line SNT8 (provided by Beyond Technology Co, xiamen, China) and Jurkat T-cells (ATCC, TIB-152, USA) were cultured in RPMI-1640 medium (Therm Fisher Scientific, 11875093, USA) supplemented with 10% FBS (Gibco, 16000044, USA) and 1% penicillin-streptomycin (Therm Fisher Scientific, 15140122, USA) under constant conditions of 37 °C and 5% CO_2_. SNT8 cells treated with different conditions (SNT8, shControl, shSmo, shSmo + Smo activator, shControl + Smo inhibitor) were co-cultured with Jurkat cells at a 1:1 ratio for 48 h. An anti-PD-1 antibody (10 µg/mL, BiLegend, 329905, USA) was added to the co-culture system.

### Cell transfection

We used the LV-*Smo*-RNAi lentiviral construct synthesized by GeneChem (China) for cell transfection. This construct carries the GFP fluorescent protein gene and the puromycin resistance gene. The shRNA sequence is 5’-gaATCGCTACCCTGCTGTTAT-3’, and the control sequence is 5’-TTCTCCGAACGTGTCACGT-3’. The plasmid vector used is GV493. SNT8 cells were seeded into a 6-well plate and infected with the lentivirus once the cells reached 70–80% confluence. 8 µg/mL polybrene (PlyBrene, Sigma-Aldrich, USA) was added to enhance infection efficiency. After 24 h, the medium was replaced with fresh medium, and 1 µL/mL puromycin (HyClone, USA) was added to select stably transfected cell lines. Transfection efficiency was observed through GFP expression using a fluorescence microscope (Olympus, Japan), Smo mRNA levels were detected using qRT-PCR (ABI-7500 Fast Real-Time PCR system, Applied Biosystems, USA), and Smo protein expression was confirmed by WB (Bio-Rad, USA).

### Fluorescence microscopy

Transfected SNT8 cells control SNT8 cells, and *Smo*-silenced SNT8/KHYG-1 cells were seeded into 12-well plates. Once the cells adhered and grew, GFP expression in the cells was observed under a fluorescence microscope (Olympus, Japan). Cell images were captured in both bright-field and green fluorescence fields to confirm transfection efficiency. Multiple fields of view were selected for imaging for each sample, ensuring consistent imaging locations to maintain comparability of the images.

### qRT-PCR

Total RNA was extracted from SNT8/KHYG-1 cells using Trizol reagent (Invitrogen, USA). The extracted RNA was reverse-transcribed into cDNA using the HiScript^®^ II 1 st Strand cDNA Synthesis Kit (Vazyme Biotech, China). qPCR was then performed using *Smo*-specific primers (Forward: 5′-CAAGATCAACGAGACCAT-3′; Reverse: 5′-CTGAAGGTAATGAGCACAA-3′), PD-L1 primers (Forward: 5′-AAGGCCGAAGTCATCTGGAC-3′; Reverse: 5′-CCAGAGGTAGTTCTGGGATGAC-3′), The qPCR detection was performed using SYBR Green PCR Master Mix (Vazyme Biotech, China) on an ABI-7500 Fast Real-Time PCR system (Applied Biosystems, USA). 18 S rRNA was used as the internal reference gene (Forward: 5′-CAGCCACCCGAGATTGAGCA-3′; Reverse: 5′-TAGTAGCGACGGGCGGTGTG-3′). The PCR conditions were set: initial denaturation at 95 °C for 10 min, followed by 40 cycles of 95 °C for 15 s and 60 °C for 1 min. The relative gene expression was calculated using the 2^−ΔΔCt^ method, and statistical analysis was performed using data from three independent experiments.

### Single-cell soft agar colony formation assay

The human NKTCL cell line SNT8/KHYG-1 (provided by leeyond technology Co, xiamen, China), sh Control SNT8/KHYG-1 cells, and *Smo*-silenced SNT8/KHYG-1 cells were adjusted to a density of 2 × 10^4^ cells/mL. In a 6-well plate, the base layer of agar was prepared by heating 0.6% agar (Difco, USA, 214220) in RPMI-1640 medium to 100 °C, cooling it to about 40–45 °C, and mixing in 10% FBS (Gibco, USA, 16000-044). Then, 2 mL of the base agar mixture was added to each well and allowed to solidify. For the top layer, 0.3% agar (Difco, USA, 214220) in RPMI-1640 medium was similarly heated, cooled to 40–45 °C, and mixed with 10% FBS (Gibco, USA, 16000-044) and treated SNT8/KHYG-1 cells. Then, 2 mL of the top agar mixture was gently layered over the solidified base agar in each well. The cells were cultured in an incubator at 37 °C with 5% CO_2_ for 2–3 weeks until colonies were visible to the naked eye. At the end of the culture period, the colonies were stained with 0.005% methylene blue (Sigma-Aldrich, USA, M9140) for 1 h and gently washed with distilled water to remove excess stain. The number of colonies was counted under an inverted microscope (Leica, Germany, DMIL LED), with multiple fields of view photographed for each well, and the number of colonies in each group was recorded.

The colony formation rate was calculated using the following formula:

 

$$\begin{aligned} \mathrm{Colony}\;\mathrm{formation}\;\mathrm{rate}(\%)=&(\mathrm{Number}\;\mathrm{of}\;\mathrm{colonies}\;\mathrm{formed}/\\&\mathrm{Total}\;\mathrm{number}\;\mathrm{of}\;\mathrm{seeded}\;\mathrm{cells})\times100 \% \end{aligned}$$

### Cell proliferation assay (CCK-8)

SNT8/KHYG-1 cells, control SNT8/KHYG-1 cells, and Smo-silenced SNT8/KHYG-1 cells were adjusted to a density of 2 × 10^4^ cells/mL and seeded into a 96-well plate, with 200 µL of cell suspension added to each well. On days 0, 1, 2, 3, 4, and 5, 10 µL of CCK-8 solution (Dojindo, Japan) was added to each well and incubated at 37 °C for 4 hours. After incubation, absorbance was measured at 450 nm using a microplate reader (Bio-Rad, USA). Cell viability was calculated using the following formula: Cell viability (%) = (Absorbance of *Smo*-silenced SNT8 cells - Absorbance of blank control)/(Absorbance of control SNT8 cells - Absorbance of blank control) × 100%. The CCK-8 assay evaluates the effect of Smo gene silencing on the proliferation capacity of SNT8 cells. The experiment was repeated three times and statistically analyzed to ensure data reliability.

### Flow cytometry

SNT8 cells, shControl SNT8 cells, and *Smo*-silenced SNT8 cells were collected and washed twice with PBS buffer, and the cell concentration was adjusted to 5 × 10^5^ to 1 × 10^6^ cells/mL. According to the manufacturer’s instructions, cell apoptosis was assessed using the annexin V-PE/7-AAD double staining (annexin V-PE/7-AAD) kit (BD Biosciences, USA). A 100 µL cell suspension was added to flow cytometer tubes containing 500 µL of binding buffer, followed by the addition of 5 µL of Annexin V-PE and 5 µL of 7-AAD. The mixture was gently vortexed and incubated in the dark at room temperature for 15 min. After incubation, the cells were analyzed using a flow cytometer (BD FACSCanto II, BD Biosciences, USA) within 1 h, and the results were analyzed using FlowJo software (Tree Star, USA).

After 48 h of co-culture, cells were collected and labeled with anti-CD69 and anti-CD25 antibodies (BiLegend, USA) to mark T-cell activation. The expression of these activation markers was detected using a flow cytometer (BD FACSCanto II, BD Biosciences, USA).

After 48 h of co-culture, the suspended cells in the co-culture system were collected and centrifuged at 1200 rpm for 5 min. The supernatant was discarded, and the cells were washed twice with 1 mL cold PBS each time, followed by centrifugation to remove any residual medium and suspended particles. The washed cells were resuspended in 100 µL PBS. Appropriate amounts of anti-CD69-PE and anti-CD25-FITC antibodies (diluted to the final concentration recommended by the manufacturer) were added, gently mixed and incubated in the dark at 4 °C for 30 min. The fluorescence intensity of CD69 and CD25 in T-cells was then detected using a flow cytometer (BD FACSCanto II, BD Biosciences, USA).

A 5 mM CFSE stock solution was prepared in serum-free PBS and diluted to a final working concentration of 1 µM using the CFSE Cell Proliferation Kit (Invitrogen, USA, C34554) for cell labeling. Logarithmic-phase Jurkat T-Cell were collected, washed twice with serum-free PBS, and adjusted to a concentration of 1 × 10⁶ cells/mL. A 1 mL cell suspension (1 × 10⁶ cells) was mixed with 1 mL of 1 µM CFSE working solution, gently mixed, and incubated in the dark at room temperature for 10 min. The staining reaction was terminated by adding 5 mL of RPMI-1640 medium containing 10% FBS, gently mixed, and centrifuged at 1200 rpm for 5 min. The supernatant was discarded, and the cells were washed twice with RPMI-1640 medium containing 10% FBS. The cell concentration was then adjusted to 1 × 10^6^ cells/mL. The fluorescence intensity of CFSE in T-Cell was detected using a flow cytometer (BD FACSCanto II, BD Biosciences, USA). The fluorescence intensity of CFSE decreases as cell division occurs, allowing the assessment of T-cell proliferation by detecting changes in fluorescence intensity.

After 48 h of co-culture, cells were collected and fixed with 70% cold ethanol at −20℃ overnight to permeabilize the membrane for dye entry. A cell apoptosis detection kit (#AD10, DOJINDO) was used. Cells were stained with propidium iodide (PI) solution containing RNase A (50 µg/mL, #KGA-511, Keygen Biotech, Jiangsu, China) and PI (50 µg/mL) for 30 min in the dark. DNA content was analyzed using a flow cytometer (BD FACSCanto II, BD Biosciences, USA) to assess cell cycle distribution (G0/G1, S, and G2/M phases), and the percentage of cells in each phase was calculated (Cao et al., 2025, (Bordoni et al. 2021; Kozak 2025) Du et al.^2017^.

### Western blot (WB)

Total protein was extracted from SNT8/KHYG-1 cells, control SNT8/KHYG-1 cells, and Smo-silenced SNT8/KHYG-1 cells using RIPA lysis buffer (Beyotime, China). The supernatant was collected after centrifugation at 20,000 g/min for 10 min at 4 °C. Protein concentration was measured using a BCA protein assay kit (Beyotime, China). For each sample, 30 µg of protein was separated by SDS-PAGE and transferred onto a PVDF membrane (Millipore, USA). The membrane was blocked with TBST buffer containing 5% non-fat milk for 1 h. Subsequently, the primary antibodies, anti-Smo (1:1000, Abcam, UK, ab236465), anti-Gli1 (1:1000, Cell Signaling Technology, USA, 2534), anti-PD-L1 (1:1000, Cell Signaling Technology, USA, 13684), anti-GAPDH (1:5000, Beyotime, China, 9662), anti-Caspase-3 (Cell Signaling Technology, USA, 9664), anti-cleaved Caspase-3 (Cell Signaling Technology, USA, 9661 S), anti-Bax (Cell Signaling Technology, USA, 2772), anti- Bcl-2(B-cell lymphoma 2) (Cell Signaling Technology, USA, 2870), anti-GAPDH (Beyotime, China, AF1186), anti-CALR (Cell Signaling Technology, USA, #12238), anti-phospho-STAT3 (P-STAT3; Cell Signaling Technology, USA, #9145), anti-PHLPP2 (Abcam, UK, ab153918), anti-phospho-AKT (P-AKT; Cell Signaling Technology, USA, #4060), and anti-MAPK (Abcam, UK, ab308333) were incubated overnight at 4 °C. The following day, the membrane was incubated with HRP-conjugated secondary antibodies (Beyotime, China) at room temperature for 1 h. Signal detection was performed using an enhanced chemiluminescence (ECL) kit (Thermo Fisher Scientific, USA), and protein bands were visualized using an imaging system (Bio-Rad, USA).

### In vivo tumor xenotransplantation growth assay

Four- to six-week-old NCG mice (ND/ShiLtJGpt-Prkdcem26Cd52Il2rgem26Cd22/Gpt) were purchased from GemPharmatech Co., Ltd. (Nanjing, China) and housed under pathogen-free conditions, with free access to clean food and water. SNT8 cells control SNT8 cells, and *Smo*-silenced SNT8 cells (2 × 10⁶ cells per mouse) were subcutaneously injected into the flanks of NCG mice, with five mice in each group. Tumor volume and mouse body weight were measured regularly, and the mice were sacrificed on day 25 to collect tumor tissues. Tumor volume was calculated using the formula: Volume (mm³) = 0.5 × Length (mm) × Width (mm)². The collected tumor tissues were weighed and subjected to HE and immunohistochemical staining to assess tumor growth and Smo protein expression. The experiment was approved by the Ethics Committee of the First Hospital of Quanzhou, Fujian Medical University. All experimental procedures conformed to the Helsinki Declaration, and written informed consent was obtained from all participants.

### Immunohistochemistry

After the mice were sacrificed, the tumor tissues were collected and fixed in 4% paraformaldehyde for 24 h. Following gradient dehydration with ethanol and clearing with xylene, the tissues were embedded in paraffin. The paraffin-embedded tumor tissues were sectioned into 4 μm-thick continuous slices using a rotary microtome (Leica, Germany, RM2235) and mounted on polylysine-coated slides, then dried overnight at 37 °C. The sections were immersed in xylene for 10 min (twice) to remove the paraffin. The sections were rehydrated by soaking in 100%, 95%, 80%, and 70% ethanol for 5 min each, then washing in distilled water for 10 min. To block endogenous peroxidase activity, the sections were incubated in 3% hydrogen peroxide (H_2_O_2_) solution for 10 min and washed three times with PBS, 5 min each. Antigen retrieval was performed by placing the sections in 10 mM sodium citrate buffer (pH 6.0) and heating them in a microwave until boiling, then maintaining the temperature for 15 min. After cooling to room temperature, the sections were washed thrice with PBS for 5 min each. The sections were then incubated with PBS containing 5% goat serum for 1 h to block non-specific antibody binding sites. After blocking, the sections were incubated overnight (4 °C) with the following specific primary antibodies: Smo(Thermo Fisher Scientific Inc, USA, 66851-1-IG, dilution 1:2000); Gli1 (Abcam, USA, ab92611, dilution 1:200); PD-L1 (Cell Signaling Technology, USA, 13684, dilution 1:100); Caspase-3 (Cell Signaling Technology, USA, 9664, dilution 1:300); and Ki67 (Abcam, USA, ab15580, dilution 1:400). The sections were washed three times with PBS for 5 min each, then incubated with HRP-labeled secondary antibodies (goat anti-rabbit or goat anti-mouse, dilution 1:200) at room temperature for 1 h. After washing with PBS, DAB chromogen solution (Dako, Denmark, K3468) was added, and the staining time was controlled between 2 and 10 min, depending on the intensity of the color development. Once staining was complete, the sections were immediately rinsed with tap water to stop the reaction. Nuclei were counterstained with hematoxylin for 1 min, then rinsed with tap water to remove excess stain. After gradient dehydration with ethanol and clearing with xylene, the sections were sealed with neutral balsam. The sections were observed under a light microscope (Olympus, Japan, BX53), and the percentage of positively stained cells was quantified using ImageJ software. Photographs of multiple high-power fields (400×) were taken for each section, and the percentage of positive cells and staining intensity were recorded. The scoring criteria were as follows: the proportion of positive cells (0-100%) multiplied by the staining intensity (0–3 points, 0 for no staining, 1 for weak positivity, 2 for moderate positivity, 3 for strong positivity), resulting in an immunoreactivity score (IRS).

### Statistical analysis

All experimental data were analyzed using GraphPad Prism 8.0 software (GraphPad Software, USA). Data are presented as mean ± standard error (SE). One-way analysis of variance (ANOVA) was used for comparisons between multiple groups, followed by least significant difference (LSD) post-hoc tests. Statistical significance was set at *p <* 0.05. * indicates a significant difference compared to the SNT8 group, # indicates a significant difference compared to the shCntrl group, and & indicates a significant difference compared to the shSmo group.

## Results

### Smo gene silencing significantly inhibits NKTCL cell proliferation

**Fig. 1:**
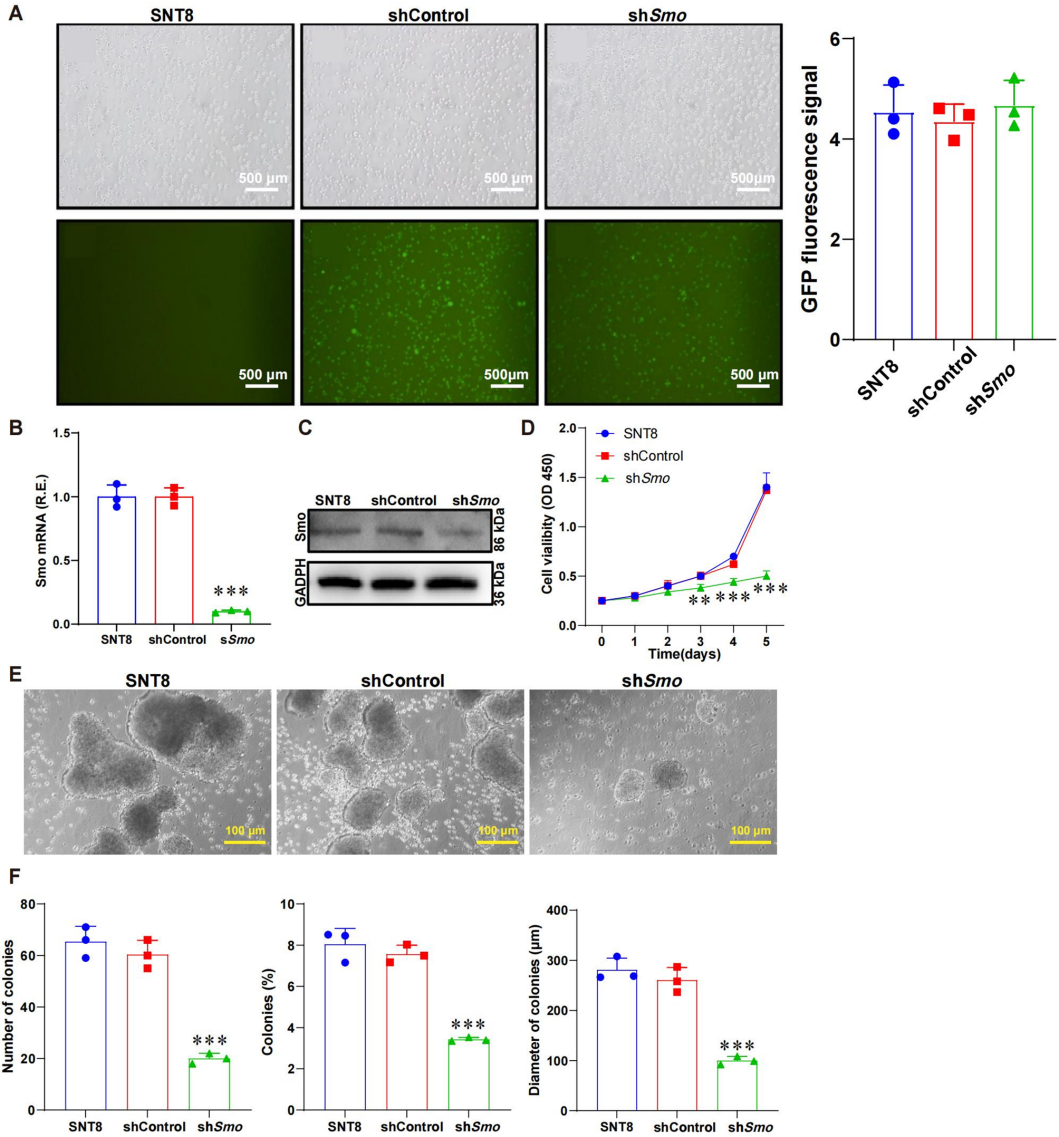
Effects of *Smo* gene silencing on NKTCL cell proliferation and apoptosis.**A** Fluorescence microscopy images of SNT8 cells, control SNT8 cells, and Smo-silenced SNT8 cells. The top row shows bright-field images, while the bottom row shows the corresponding green fluorescence images (400×), bar = 500 μm; the right panel quantifies GFP signal intensity. **B** qRT-PCR detection of *Smo* mRNA expression levels in SNT8 cells, control SNT8 cells, and Smo-silenced SNT8 cells; **C** WB analysis of *Smo* protein expression in SNT8 cells, control SNT8 cells, and Smo-silenced SNT8 cells. GAPDH is used as the internal reference; **D** CCK-8 assay for the detection of proliferation in SNT8 cells, control SNT8 cells, and Smo-silenced SNT8 cells; **E** Microscopic images showing the spheroid formation of cells; **F** The number of spheroids, spheroid formation rate, and spheroid diameter in NKTCL cells. Statistical analysis was performed using one-way ANOVA, and data are presented as mean ± SE, *n* = 3. *Indicates a significant difference compared to the shCntrl group. Significant differences are indicated as * *p <* 0.05, ** *p <* 0.01, *** *p <* 0.001. R.E.: Relative expression

SNT8 cells were transduced with LV-*Smo*-RNAi lentivirus using GFP as a marker. Brightfield images showed normal morphology and strong GFP fluorescence, indicating high transduction efficiency (1A, S1A). To verify knockdown efficiency, qRT-PCR and Western blot were performed. *Smo* mRNA and protein levels were markedly reduced in the sh*Smo* group compared to controls (Fig. 1B-C, S1B-C). To assess proliferation, CCK-8 assays were conducted over five days. The sh*Smo* group showed significantly reduced proliferation compared to SNT8 and shControl cells (Fig. 1D, S1D). To evaluate clonogenic potential, soft agar colony formation assays were performed. The sh*Smo* group showed a significant decrease in colony number and size (*p* < 0.05), with irregular morphology, lower density, and partial disintegration (Fig. 1E-F).T.

### Smo gene silencing promotes snt8 cell apoptosis

**Fig. 2:**
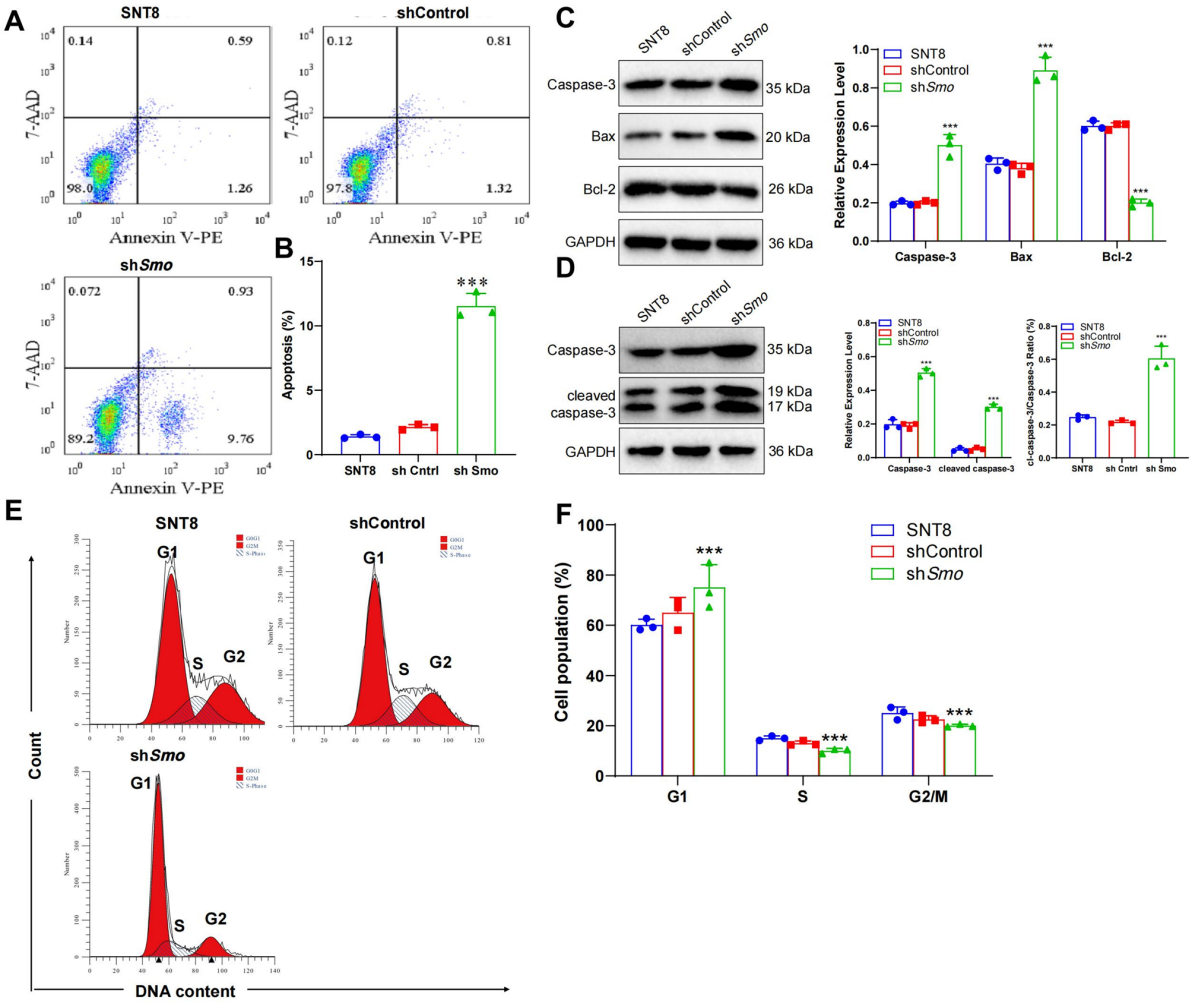
*Smo* gene silencing promotes apoptosis in NKTCL cells. **A** Annexin V-PE and 7-AAD double staining were used to detect the apoptosis rate of SNT8 cells, control SNT8 cells, and Smo-silenced SNT8 cells by flow cytometry. The lower left quadrant represents viable non-apoptotic cells, the lower right quadrant represents early apoptotic cells, the upper left quadrant represents mechanically damaged cells, and the upper right quadrant represents late apoptotic or dead cells; **B** Statistical graph showing the proportion of apoptotic cells in SNT8 cells, control SNT8 cells, and Smo-silenced SNT8 cells; **C** WB analysis of changes in protein expression levels of Caspase-3, Bax, and Bcl-2; **D** WB analysis of the expression levels of Caspase-3, cleaved Caspase-3, and the ratio of cleaved Caspase-3 to Caspase-3. **E** Cell cycle distribution of SNT8, shCtrl SNT8, and shSmo SNT8 cells analyzed by PI staining and flow cytometry. **F** Quantification of cell cycle phase proportions in each group. Data are presented as mean ± SE, *n* = 3. *Indicates a significant difference compared to the shCntrl group. Significant differences are indicated as * *p <* 0.05, ** *p <* 0.01, *** *p <* 0.001.Statistical analysis was performed using one-way ANOVA

Apoptosis was evaluated in SNT8 cells following LV-*Smo*-RNAi transduction using Annexin V-PE/7-AAD staining and flow cytometry. The sh*Smo* group showed a marked increase in early and late apoptotic cells compared to the SNT8 and shControl groups (10.69% vs. 1.85% and 2.13%, respectively; Fig. 2A, S1E). Quantification confirmed a significantly elevated apoptosis rate in the sh*Smo* group (Fig. 2B, S1F). Western blot analysis revealed increased expression of Caspase-3 and Bax, along with reduced Bcl-2 levels in sh*Smo* cells relative to shControl cells (Fig. 2C, S1I). The densitometric analysis supported these changes. Cleaved Caspase-3 was further examined to assess proteolytic activation. A significant increase in cleaved Caspase-3 and the cleaved/full-length Caspase-3 ratio was observed in the sh*Smo* group (Fig. 2D, S1J). Cell cycle analysis showed G1 phase accumulation and a corresponding decrease in S and G2/M phases in sh*Smo* cells, indicating potential G1 arrest (Fig. 2E, S1G-H).

### The critical role of Smo in the regulation of NKTCL cell proliferation and apoptosis

**Fig. 3:**
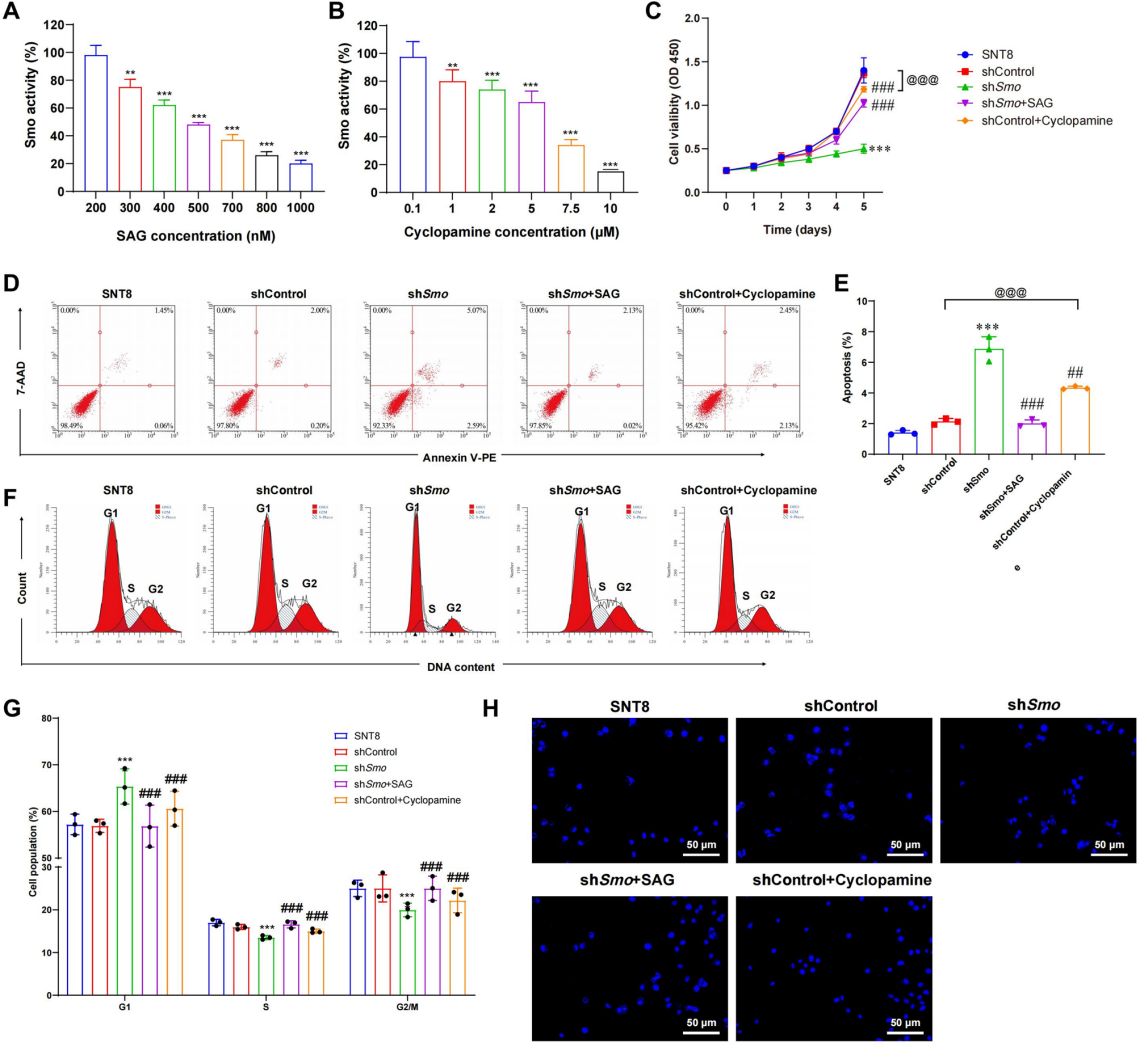
Effects of *Smo* activator and inhibitor on NKTCL cell proliferation and apoptosis. **A, B** *Smo* activity following treatment with different concentrations of SAG (A) or Cyclopamine (B), *Smo* activity (%) is shown relative to untreated SNT8 cells (set as 100%). **C** Cell proliferation assessed by CCK-8 assay. **D, E** Apoptosis analysis by flow cytometry, including quantification of apoptotic rates after SAG or Cyclopamine treatment. **F** Cell cycle distribution of SNT8, shCtrl SNT8, and shSmo SNT8 cells analyzed by PI staining. **G** Quantification of cell cycle phase distribution. **H** Nuclear morphology observed by DAPI staining. Data are presented as mean ± SE, *n* = 3. *Indicates a significant difference compared to the shCntrl group, with significant differences expressed as * *p <* 0.05, ** *p <* 0.01, *** *p <* 0.001. #Indicates a significant difference compared to the sh *Smo* group, with significant differences expressed as # *p <* 0.05, ## *p <* 0.01, ###*p <* 0.001. Statistical analysis was performed using one-way ANOVA

SNT8 cells were treated with the Smo agonist SAG and the inhibitor Cyclopamine to determine optimal concentrations. SAG at 200 nM and Cyclopamine at 10 µM were selected (Fig. 3A, B). sh*Smo* cells were treated with SAG and shControl cells with Cyclopamine. CCK-8 assays showed that SAG-treated shSmo cells exhibited proliferation levels comparable to SNT8 and shControl (*p* > 0.05), while Cyclopamine treatment reduced proliferation in shControl cells (*p* < 0.05; Fig. 3C, S2A). Apoptosis was assessed by flow cytometry. SAG-treated sh*Smo* cells showed reduced early and late apoptosis, similar to shControl cells (*p* > 0.05), whereas Cyclopamine increased apoptosis in shControl cells (*p* < 0.05; Fig. 3D, S2B). Quantification showed an apoptosis rate of 2.5% in SAG-treated sh*Smo *cells, compared to 1.40% in SNT8, 2.13% in shControl, and 7.02% in untreated sh*Smo*. Cyclopamine-treated shControl cells reached 4.75% (Fig. 3E, S2C). Cell cycle analysis revealed G1 phase accumulation in sh*Smo* cells. SAG treatment reduced the G1 fraction in sh*Smo* cells. In contrast, Cyclopamine increased G1 arrest in shControl cells, with corresponding decreases in S and G2/M phases (*p* < 0.001; Fig. 3F, G, S2D, E). Nuclear morphology was evaluated by DAPI staining. Nuclear fragmentation was observed in sh*Smo* cells. SAG-treated sh*Smo* cells showed morphology similar to shControl cells. Cyclopamine-treated shControl cells showed increased nuclear fragmentation (Fig. 3H, S2F).

### Smo gene silencing affects NKTCL cell drug resistance

**Fig. 4:**
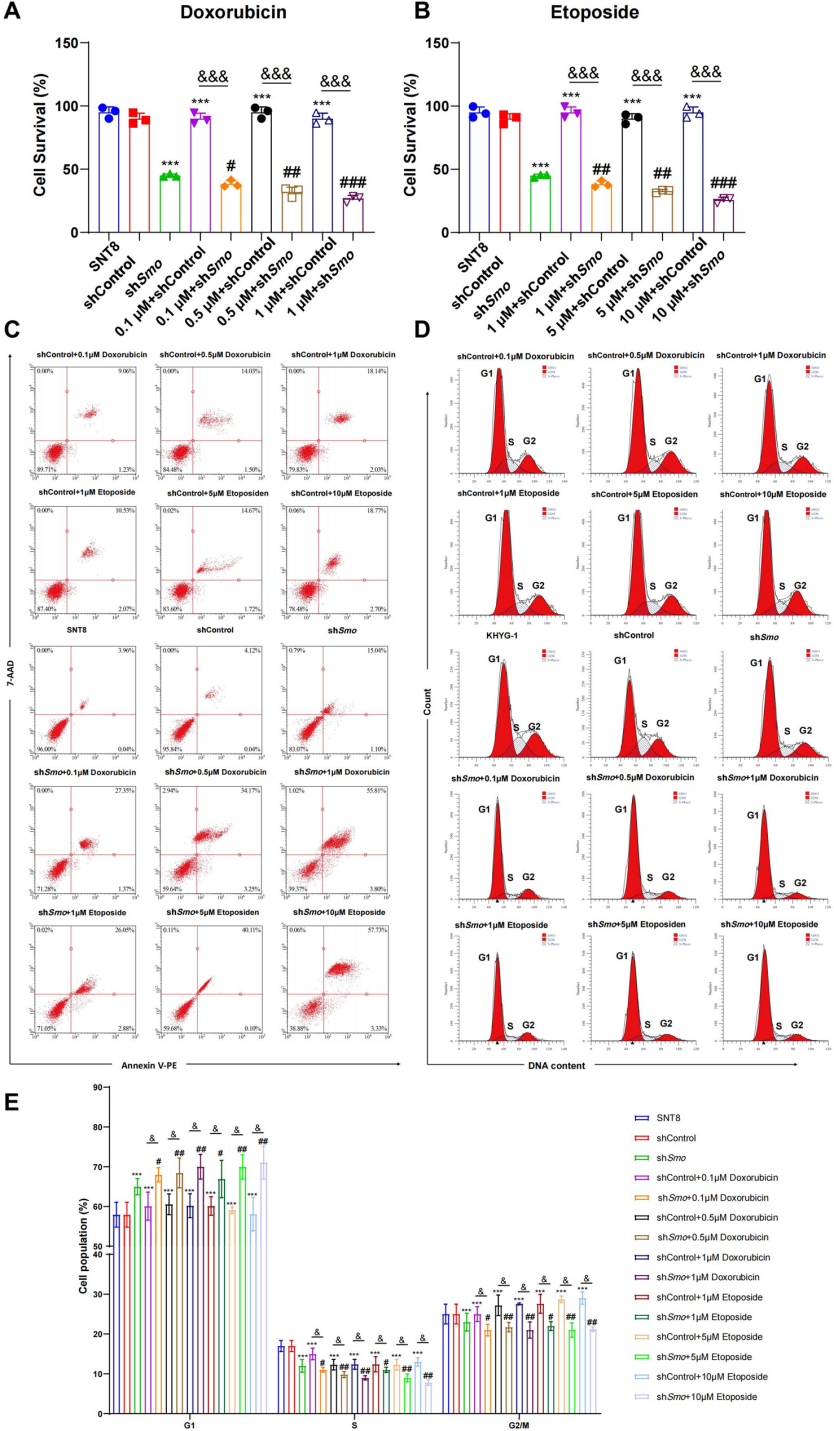
Effects of *Smo* downregulation on drug resistance in NKTCL cells.** A**, **B** Cell viability following treatment with Doxorubicin (A) or Etoposide (B), assessed by CCK-8 assay. **C** Apoptosis analysis by flow cytometry. **D** Cell cycle distribution of SNT8, shCtrl SNT8, and shSmo SNT8 cells analyzed by PI staining and flow cytometry. **E** Quantification of cell cycle phase distribution across groups. Data are presented as mean ± standard error (SE), *n* = 3. Statistical analysis was performed using one-way ANOVA.*p < 0.05, **p < 0.01, ***p < 0.001 vs. shCtrl group; #*p* < 0.05, ##*p* < 0.01, ###*p* < 0.001 vs. shSmo group;&*p* < 0.05, &&*p* < 0.01, &&&*p* < 0.001 for comparisons between shCtrl and shSmo under the same drug concentration

**Fig. 5:**
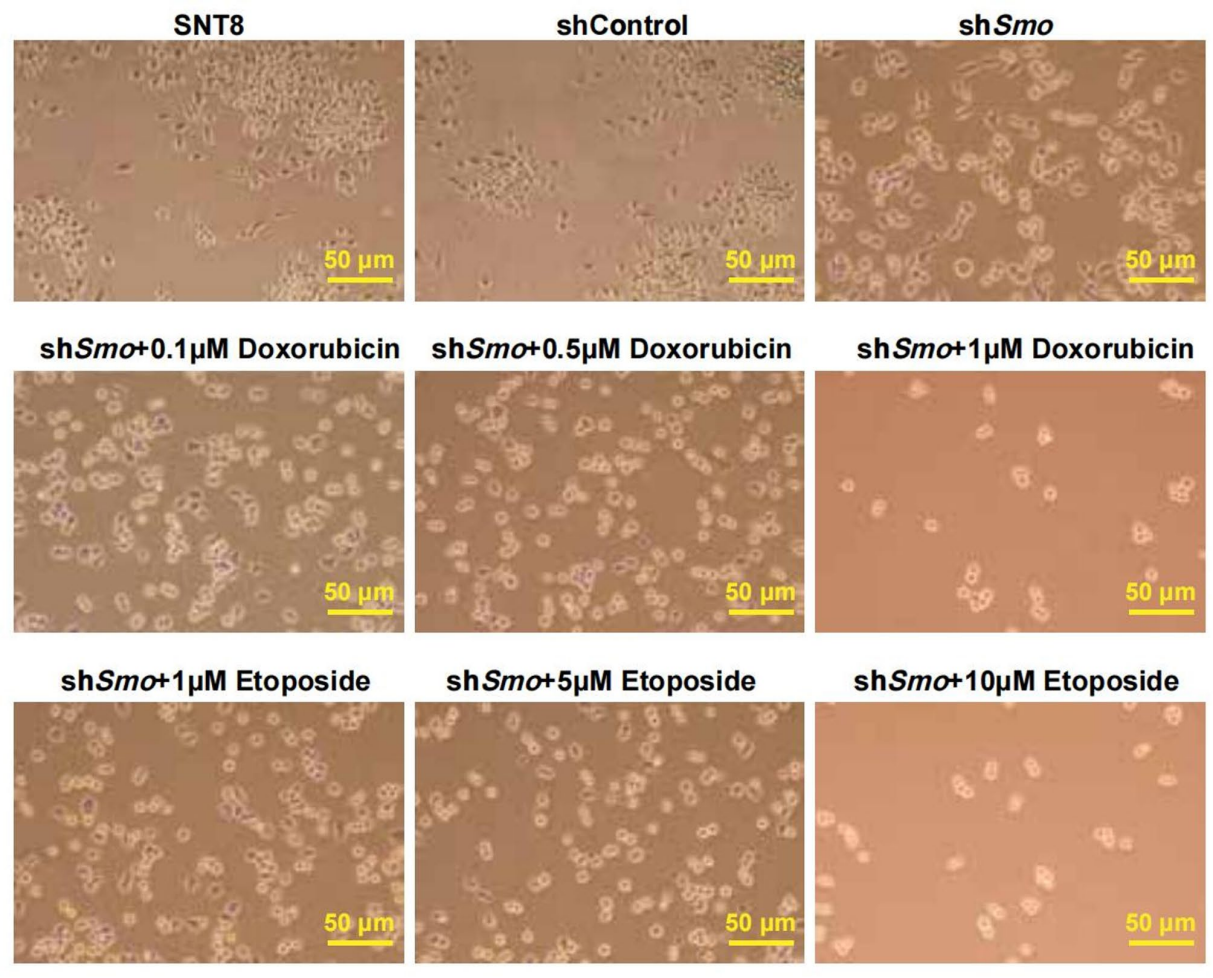
The effect of doxorubicin and etoposide treatment on cell morphology in the shSmo group. Cell morphological changes were observed using a live-cell imaging system

Drug sensitivity was evaluated by treating SNT8 cells with Doxorubicin or Etoposide. CCK-8 assays showed that cell viability decreased significantly in the sh*Smo* group upon drug treatment, with a dose-dependent reduction observed (Fig. 4A, B; S3A, B). Viability in sh*Smo* cells was significantly lower than in untreated or shControl cells (*p* < 0.05). Flow cytometry analysis further revealed increased apoptosis and G1-phase arrest in drug-treated sh*Smo *cells compared to untreated sh*Smo* cells. S and G2/M phase populations were reduced (*p* < 0.05; Fig. 4C-E, S3C-E). Doxorubicin and Etoposide alone also reduced cell survival in NKTCL cells compared with controls. Live-cell imaging showed enhanced morphological changes in sh*Smo* cells after drug treatment. Treated cells exhibited increased shrinkage, rounding, and detachment, consistent with apoptotic morphology (Fig. 5; S4).

### Smo gene silencing downregulates Gli1 and PD-L1 expression

**Fig. 6:**
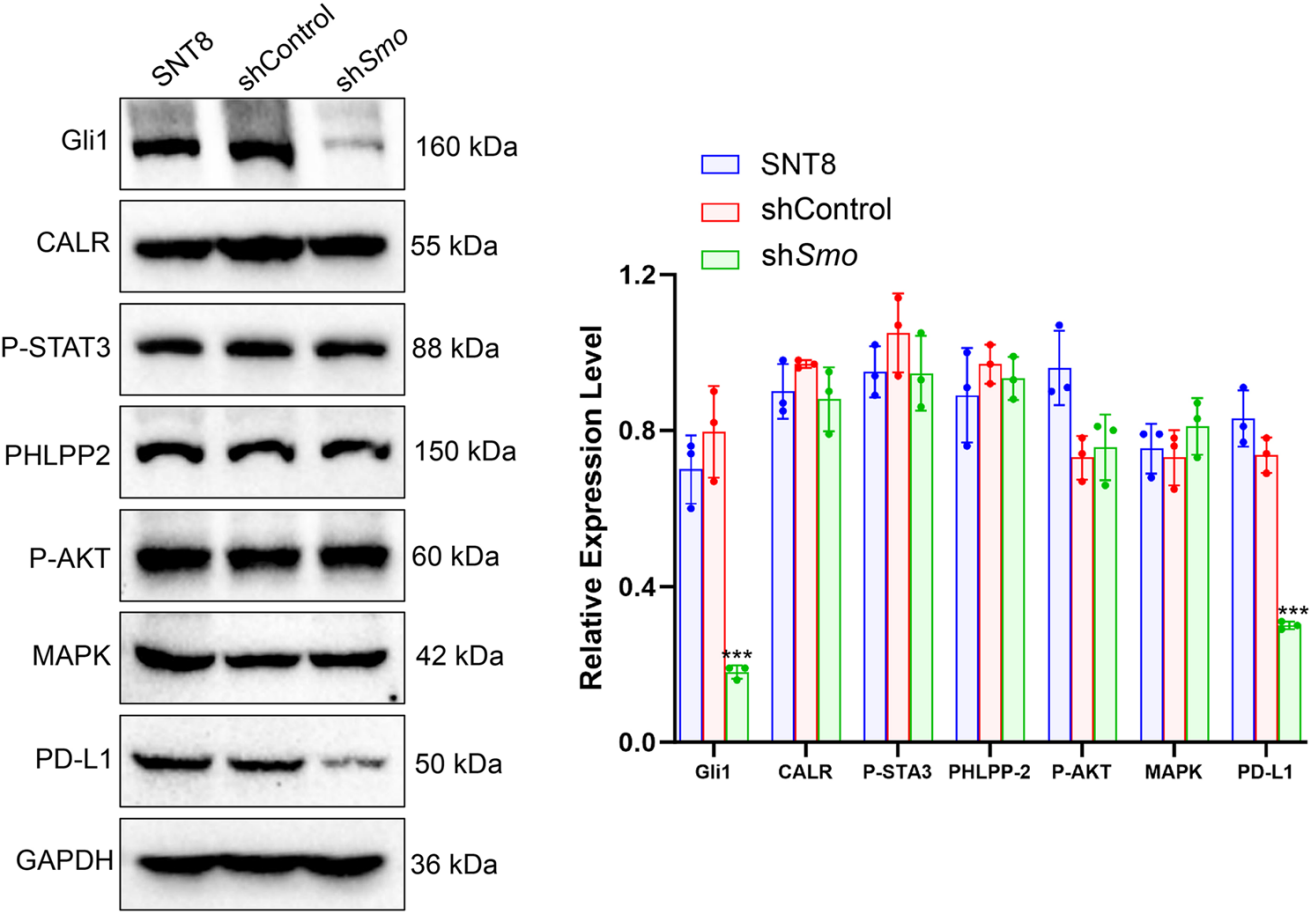
Effects of *Smo* gene silencing on the expression of proteins related to signaling pathways in NKTCL cells. WB analysis to detect the expression levels of Gli1, CALR, P-STAT3, PHLPP2, P-AKT, MAPK, and PD-L1 proteins in SNT8 cells, control SNT8 cells, and Smo-silenced SNT8 cells. GAPDH is used as an internal reference protein. Data are presented as mean ± SE, *n* = 3.*Indicates a significant difference compared to the sh *Smo* group. Significant differences are indicated as * *p <* 0.05, ** *p <* 0.01, *** *p <* 0.001. Statistical analysis was performed using one-way ANOVA

To explore downstream molecular changes associated with *Smo* silencing, Western blot was performed to assess Gli1, PD-L1, and components of the PI3K/AKT and JAK-STAT pathways in SNT8 cells. Gli1 and PD-L1 protein levels were markedly reduced in the sh*Smo* group, while CALR, PHLPP2, p-STAT3, p-AKT, and MAPK showed no significant changes (Fig. 6). Similar results were observed in KHYG-1 cells, as shown in Figure S5.

### Smo plays a key role in immune regulation by modulating PD-L1 expression and cytokine secretion

**Fig. 7:**
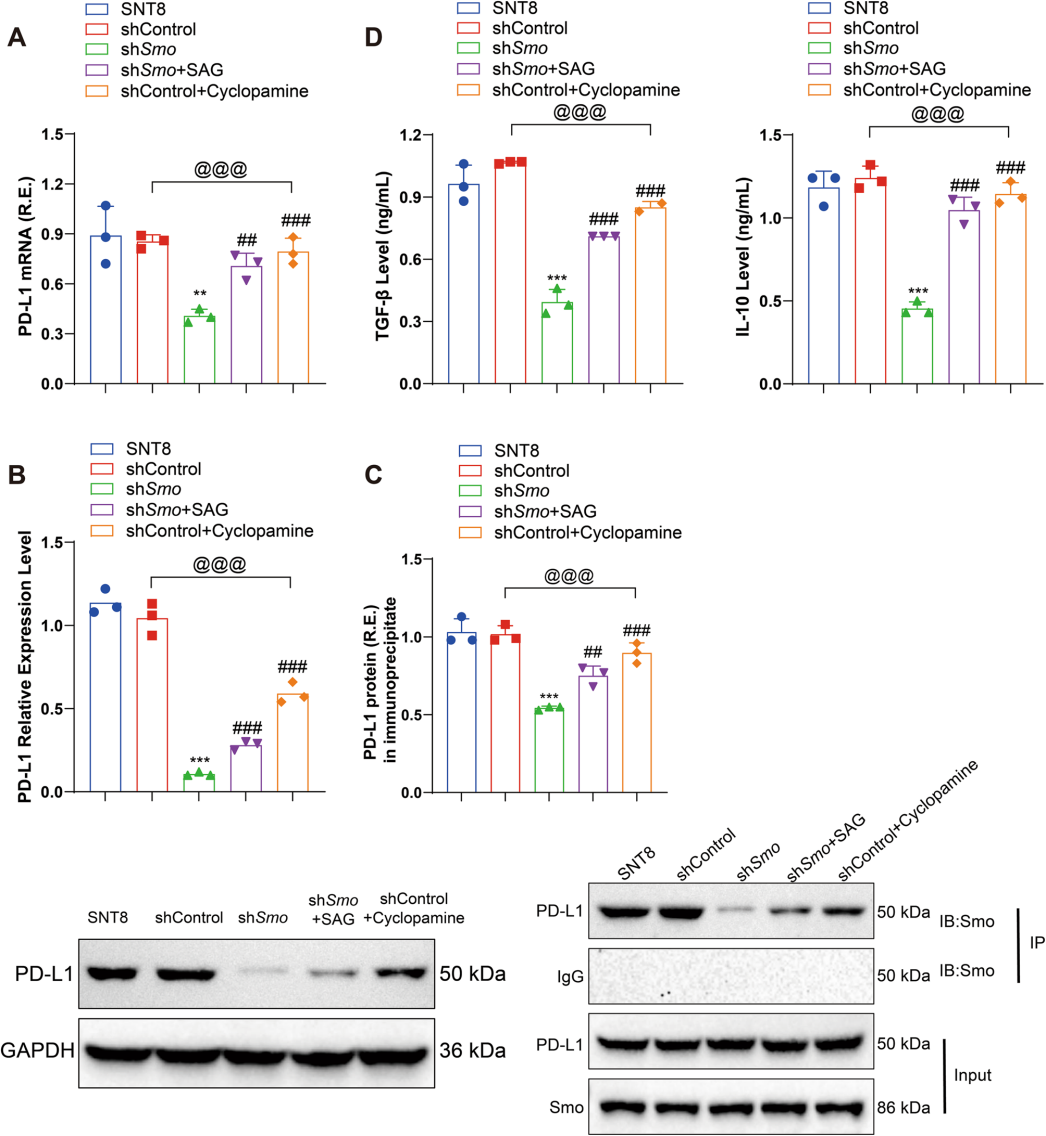
*Smo* regulates PD-L1 expression and cytokine secretion. **A** RT-qPCR analysis of changes in PD-L1 mRNA expression in cells; **B** WB analysis of changes in PD-L1 protein expression in cells; **C** Co-immunoprecipitation assay to detect the interaction between *Smo* and PD-L1; **D** ELISA analysis of the levels of TGF-β and IL-10 in the cell supernatant. Data are presented as mean ± SE, *n* = 3. *Indicates a significant difference compared to the shCntrl group, with significant differences expressed as * *p <* 0.05, ** *p <* 0.01, *** *p <* 0.001. #Indicates a significant difference compared to the sh *Smo* group, with significant differences expressed as # *p <* 0.05, ## *p <* 0.01, ###*p <* 0.001. Statistical analysis was performed using one-way ANOVA. R.E.: Relative expression

PD-L1 expression was examined following treatment with a Smo agonist (SAG) in sh*Smo* cells and a Smo inhibitor (Cyclopamine) in shControl cells. qRT-PCR showed increased PD-L1 mRNA levels in SAG-treated sh*Smo* cells, comparable to SNT8 and shControl cells, while Cyclopamine treatment reduced PD-L1 mRNA expression in shCtrl cells (*p* < 0.05; Fig. 7A, S6A). Western blot analysis showed consistent changes at the protein level: PD-L1 expression increased in SAG-treated sh*Smo* cells and decreased in Cyclopamine-treated shControl cells (*p* < 0.05; Fig. 7B, S6B). Co-immunoprecipitation analysis revealed a direct interaction between Smo and PD-L1 in shControl cells, which was reduced in sh*Smo* cells. This interaction was enhanced by SAG treatment (Fig. 7C, S6C). ELISA analysis of cell culture supernatants showed lower levels of TGF-β and IL-10 in the sh*Smo* group compared to SNT8 and shControl cells. These levels increased after SAG treatment and decreased following Cyclopamine exposure (*p* < 0.05; Fig. 7D, S6D).

### Smo plays a key role in NKTCL cell immune evasion by regulating T-cell activation, proliferation, and apoptosis

**Fig. 8:**
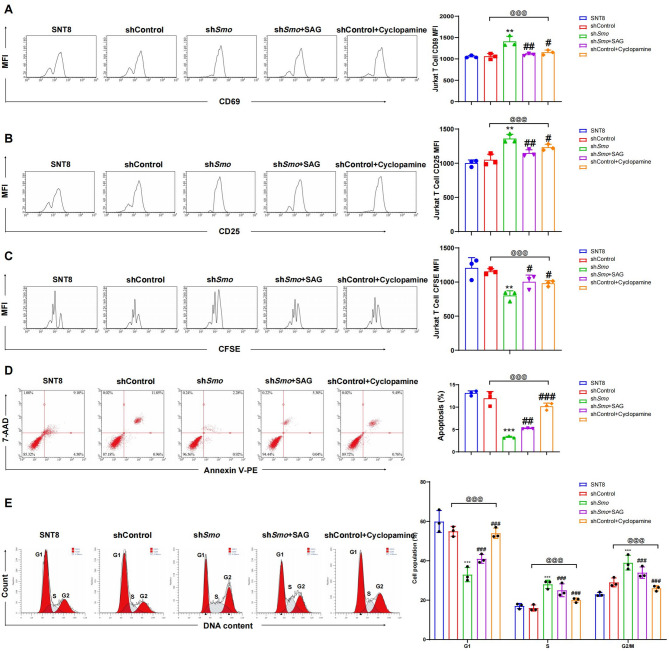
Regulatory role of *Smo* in T cell activation, proliferation, and apoptosis. **A** Flow cytometry analysis of the MFI of CD69 in T-Cell after 48 h of co-culture; **B** Flow cytometry analysis of the MFI of CD25 in T-Cell after 48 h of co-culture; **C** CFSE dye labeling to detect T cell proliferation, with MFI reflecting the number of cell divisions; **D** Annexin V-PE/7-AAD to detect early and late apoptosis rates in T-Cell. **E** Cell cycle distribution of SNT8, shCtrl SNT8, and shSmo SNT8 cells analyzed by PI staining and flow cytometry; right panel quantifies cell cycle phase proportions. Data are presented as mean ± SE, *n* = 3. *Indicates a significant difference compared to the shCntrl group, with significant differences expressed as * *p <* 0.05, ** *p <* 0.01, *** *p <* 0.001. #Indicates a significant difference compared to the sh *Smo* group, with significant differences expressed as # *p <* 0.05, ## *p <* 0.01, ###*p <* 0.001. Statistical analysis was performed using one-way ANOVA

Flow cytometry analysis after 48 h of co-culture showed that CD69 and CD25 mean fluorescence intensity (MFI) in T cells was significantly higher in the shSmo group compared to shControl. MFI decreased following SAG treatment and increased in Cyclopamine-treated shControl cells (*p* < 0.05; Fig. 8A-B, S7A-B). CFSE staining showed reduced MFI in the sh*Smo* group, indicating enhanced T cell proliferation. MFI increased with SAG treatment and decreased following Cyclopamine exposure (*p* < 0.05; Fig. 8 C, S7C). Annexin V-PE/7-AAD staining showed lower apoptosis rates in T cells co-cultured with sh*Smo* cells than shControl. SAG increased T cell apoptosis, while Cyclopamine had a similar effect in the shControl group (*p*< 0.05; Fig. 8D, S7D). Differences between SNT8 and shControl may be related to lentiviral transduction-induced stress responses (Chen et al. 2019; Kurena et al. 2017). PI staining revealed a lower proportion of T cells in G1 phase in the shSmo group relative to shCtrl. SAG treatment increased the G1 population, while Cyclopamine further elevated G1 arrest with corresponding reductions in S and G2/M phases (*p* < 0.05; Fig. 8E, S7E).

### Smo gene silencing inhibits NKTCL tumor growth in vivo

**Fig. 9:**
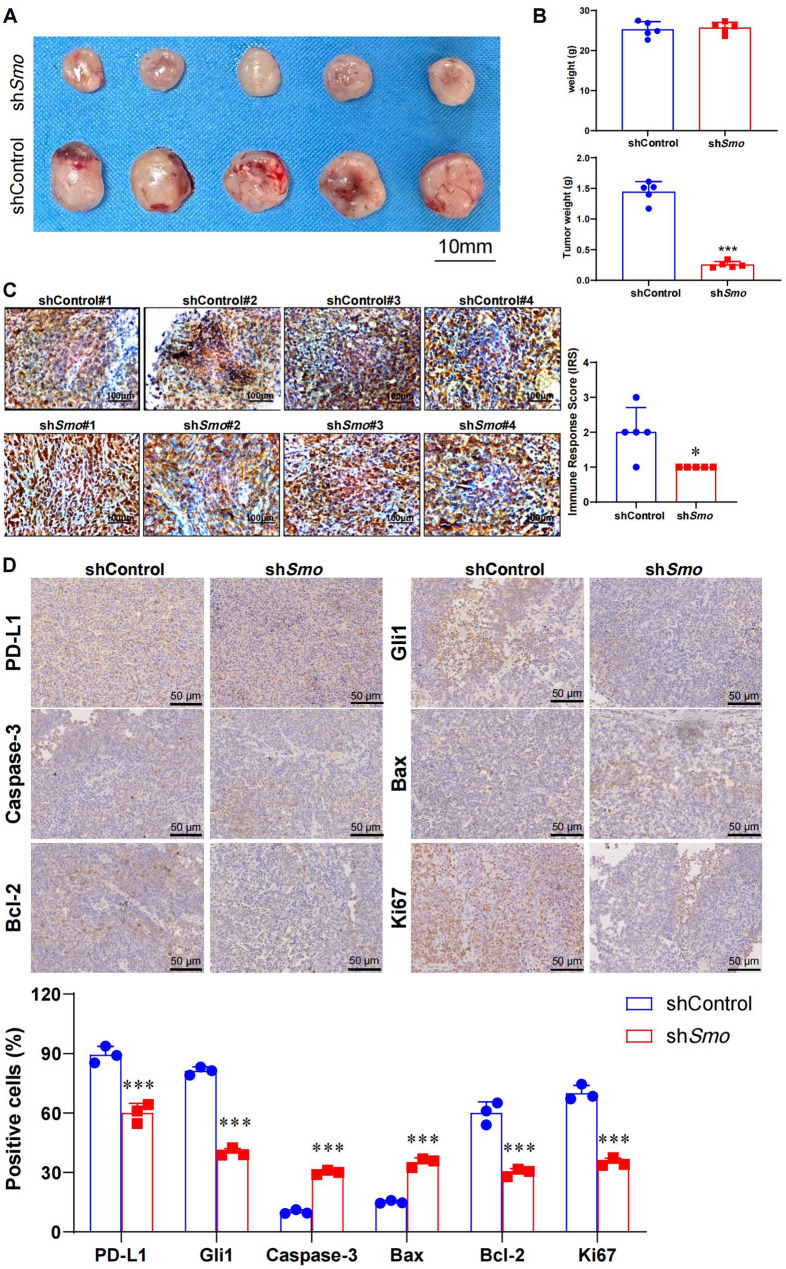
Comparison of NKTCL xenograft tumor growth in NCG nude mice. **A** Tumor appearance formed in NCG mice from the control group (shCntrl SNT8) and Smo-silenced group (shSmo SNT8); **B** Statistical analysis of the average tumor weight (g) in the control and Smo-silenced groups; **C** IHC detection of *Smo* expression in transplanted tumors in shSmo group, shCntrl group; **D** Immunohistochemical detection of Gli1, PD-L1, Caspase-3, and Ki67 protein expression levels in the tumor tissues of mice. Data are presented as mean ± SE, *n* = 5.*Indicates a significant difference compared to the shCntrl group. Significant differences are indicated as * *p <* 0.05, ** *p <* 0.01, *** *p <* 0.001. Statistical analysis was performed using one-way ANOVA

To evaluate the effect of *Smo* silencing on NKTCL tumor growth In vivo, a xenograft model was established by subcutaneously injecting 2 × 10^6^ shControl SNT8 or sh*Smo* SNT8 cells into the flanks of NCG mice (*n* = 5 per group). No deaths or significant weight changes were observed during the 25-day experimental period (Fig. 9B), indicating good tolerability. Tumor volumes in the sh*Smo* group were markedly reduced compared to the shControl group (Fig. 9 A), and final tumor weights were significantly lower (Fig. 9B). IHC confirmed reduced Smo protein expression in tumors from the sh*Smo* group, while the shControl group showed higher expression levels (Fig. 9 C; Table 1). Additional IHC analysis showed decreased expression of Gli1 and PD-L1, increased Caspase-3 and Bax, and reduced Bcl-2 in shSmo tumors compared to controls. Ki67 expression was also reduced in the sh*Smo* group (Fig. 9D), indicating reduced proliferative activity.

**Table 1:** Smo expression in NKTCL xenograft tumor

	*n*	*n*				positive(%)	*Z*	*p*
		-	**+**	**++**	**+++**			
shSmo SNT8	5	0	5	0	0	100	−2.000	0.046
shControl SNT8	5	0	1	3	1	100		

## Discussion

NKTCL is an aggressive subtype of non-Hodgkin lymphoma with poor prognosis (Yan 2023). Current treatment strategies remain limited in efficacy, particularly in cases of relapse or drug resistance (Nie 2016; Wang 2021). This highlights the need for novel molecular targets to improve therapeutic outcomes. Due to the rarity of NKTCL and the limited availability of stable human cell resources, representative cell lines are commonly used as experimental models. For instance, the SNK-6 cell line is frequently employed in xenograft models to investigate the role of tumor suppressor genes and other regulatory molecules (Xu 2022). The SNT8 cell line, characterized by high expression of genes such as MTMR2, CALR, and FOXQ1, is particularly suitable for studies on malignant progression, metastasis, and therapeutic targeting in NKTCL (Zheng 2020)Liu and (Liu 2018).

The Smo protein, as a key component of the Hedgehog signaling pathway (Zhang 2021), has garnered considerable attention in recent years. However, its specific role in NKTCL remains unclear. This study investigated the effects of Smo gene silencing on proliferation and apoptosis in NKTCL cells and evaluated its role in a xenograft mouse model. Smo knockdown significantly inhibited SNT8 cell proliferation and increased apoptosis. Expression of pathway-related proteins such as Gli1 and PD-L1 was markedly reduced. In vivo, Smo silencing suppressed tumor growth in xenograft models, supporting its role in NKTCL cell survival. These findings were further validated in the KHYG-1 cell line (de Mel et al. 2022), where Smo knockdown similarly reduced proliferation, enhanced apoptosis, and increased sensitivity to Doxorubicin and Etoposide. In KHYG-1 cells, Smo also interacted with PD-L1 and regulated immune evasion. Silencing Smo enhanced T cell activation and impaired immune escape, suggesting that the effects of Smo knockdown are broadly applicable across NKTCL models.

The role of Smo protein has been extensively studied in various tumor types, particularly in solid tumors such as melanoma and pancreatic cancer (Jeng et al. 2020). However, research on its role in NKTCL is limited. Previous literature indicates that the activation of the Smo signaling pathway is typically closely associated with tumor cell proliferation, differentiation, and apoptosis (Peng et al. 2022), and the findings of this study are consistent with these studies. This research demonstrates that Smo gene silencing inhibits cell proliferation and promotes cell apoptosis, further supporting the importance of the Smo signaling pathway in tumorigenesis. Unlike other studies, this research is the first to confirm the regulatory effect of Smo on PD-L1 expression in NKTCL cells, suggesting a potential role of Smo in immune evasion mechanisms, enriching our understanding of the role of Smo in the tumor microenvironment.

An unexpected finding in this study was the significant inhibitory effect of Smo silencing on PD-L1 expression. In most studies, PD-L1 expression is typically associated with tumor immune evasion mechanisms, where high levels of PD-L1 help tumor cells escape immune system surveillance. This study is the first to discover that Smo gene silencing can inhibit PD-L1 expression, providing new insights into the immunotherapy of NKTCL. Compared to research on other tumor types, this study reveals a potential mechanism by which Smo may influence tumor immune evasion by regulating the PD-L1 signaling pathway. This finding suggests that Smo inhibitors may not only act by directly inhibiting tumor cell proliferation but also by modulating the immune microenvironment to enhance the efficacy of immunotherapy.

In terms of scientific value, this study deeply explored the regulatory mechanisms of *Smo* in NKTCL cell proliferation and apoptosis, enhancing our understanding of the molecular control of NKTCL cell behavior. Notably, the findings reveal a connection between Smo and regulating Gli1 and PD-L1 expression, which may pave the way for developing novel anti-tumor drugs targeting these pathways.

From a clinical standpoint, since *Smo* gene silencing effectively inhibits tumor cell growth and promotes apoptosis, this strategy has the potential to be developed as a new treatment approach for NKTCL. Considering the aggressive nature of NKTCL and the limitations of current treatment options, the molecular targets identified in this study may have significant implications for improving patient prognosis.

However, the study has some limitations. First, while the in vitro results are promising, the complexity of In vivo models far exceeds that of in vitro systems, necessitating more animal studies and subsequent clinical trials to validate the applicability and effectiveness of these findings. Additionally, Smo silencing may affect multiple signaling pathways, and the interactions of these pathways and their comprehensive impact on cell behavior remain unclear, requiring further systems biology research.

Looking ahead, future studies will aim to evaluate the antitumor effects of Smo inhibitors in SNT8 xenograft models to better reflect clinical application scenarios. Further research should focus on validating the therapeutic potential of *Smo* as a target through larger-scale In vivo experiments and preclinical studies, including in other NKTCL subtypes and related malignancies. In parallel, investigations should be extended to assess how Smo silencing influences the immune microenvironment and interacts with existing treatments such as chemotherapy and radiotherapy. These efforts will help inform the development of more precise and personalized cancer treatment strategies.

## Conclusion

**Fig. 10:**
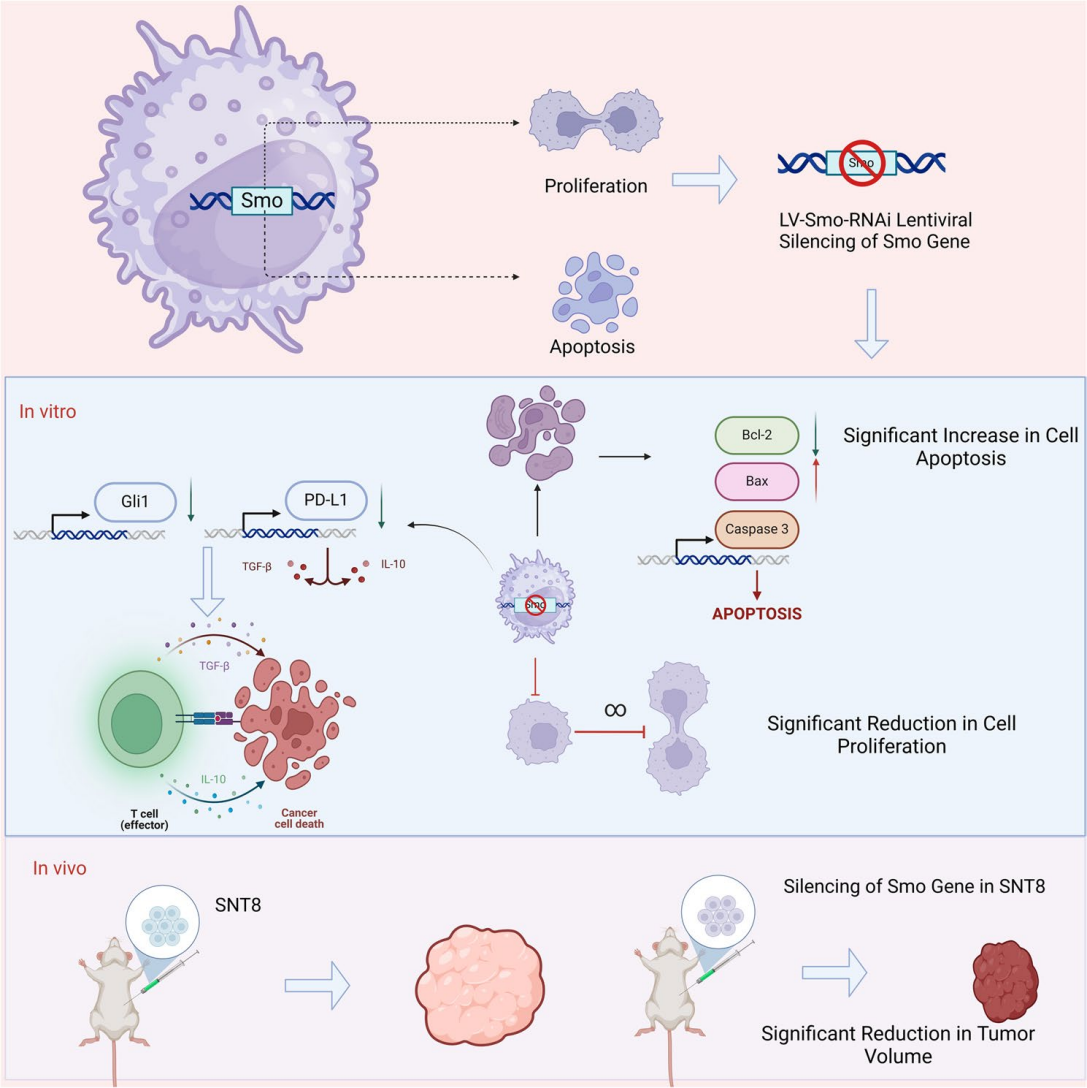
Suppressive effects of *Smo* gene silencing on NKTCL cell proliferation and apoptosis and its potential clinical application

This study, by silencing the* Smo* gene in the human NKTCL cell line SNT8, clearly demonstrated the critical role of the Smo gene in regulating cell proliferation and apoptosis. The experimental results showed that *Smo* silencing significantly inhibited the proliferation of NKTCL cells, markedly increased the apoptosis rate, and affected the expression of Gli1 and PD-L1 (Fig. 10). These findings provide new perspectives on the molecular mechanisms of NKTCL and potential therapeutic strategies.

## Supplementary information

Supplementary Material 1.

Supplementary Material 2.

Supplementary Material 3.

Supplementary Material 4.

Supplementary Material 5.

Supplementary Material 6.

Supplementary Material 7.

## Data Availability

All data can be provided as needed.

## Abbreviations

Annexin V-PE/7-AAD: Annexin V-PE/7-AAD Double Staining
ANOVA: Analysis of Variance
Bcl-2: B-cell Lymphoma 2
FBS: Fetal Bovine Serum
Gli1: GLI family Zinc Finger 1
NKTCL: Natural Killer/T-Cell Lymphoma
PD-L1: Programmed Death-Ligand 1
Smo: Smoothened
WB: Western blot
