# Supplementary figures and images for: Smo gene silencing: a promising strategy for natural killer/t-cell lymphoma treatment via modulating proliferation and apoptosis
Source: Mol Med. 2025 Oct 29;31:319. doi: 10.1186/s10020-025-01341-z (PMC12574012; doi:10.1186/s10020-025-01341-z)

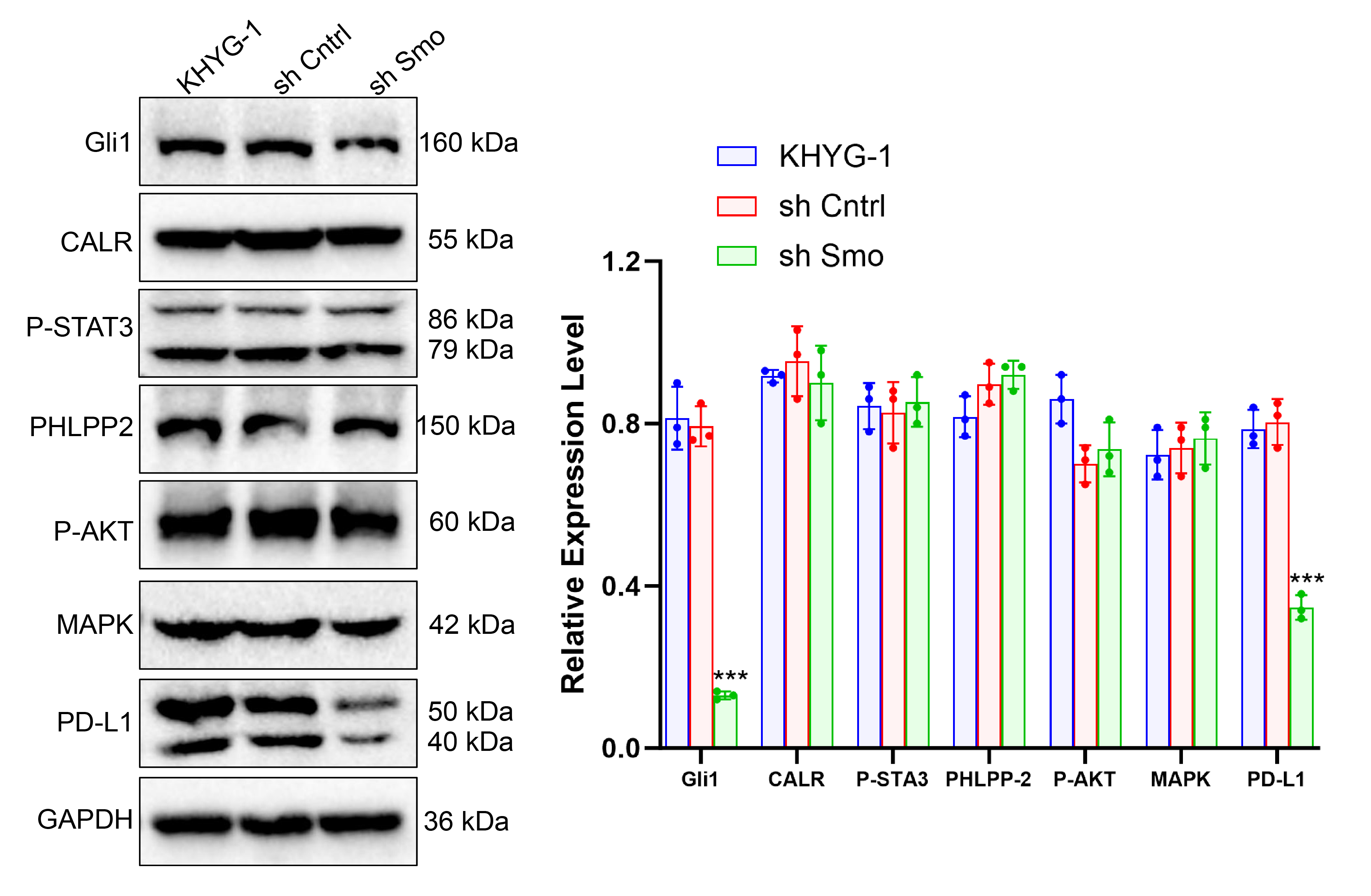

Supplement: Supplementary file 1 — Supplementary Material 1. [file 10020_2025_1341_MOESM1_ESM.jpg]

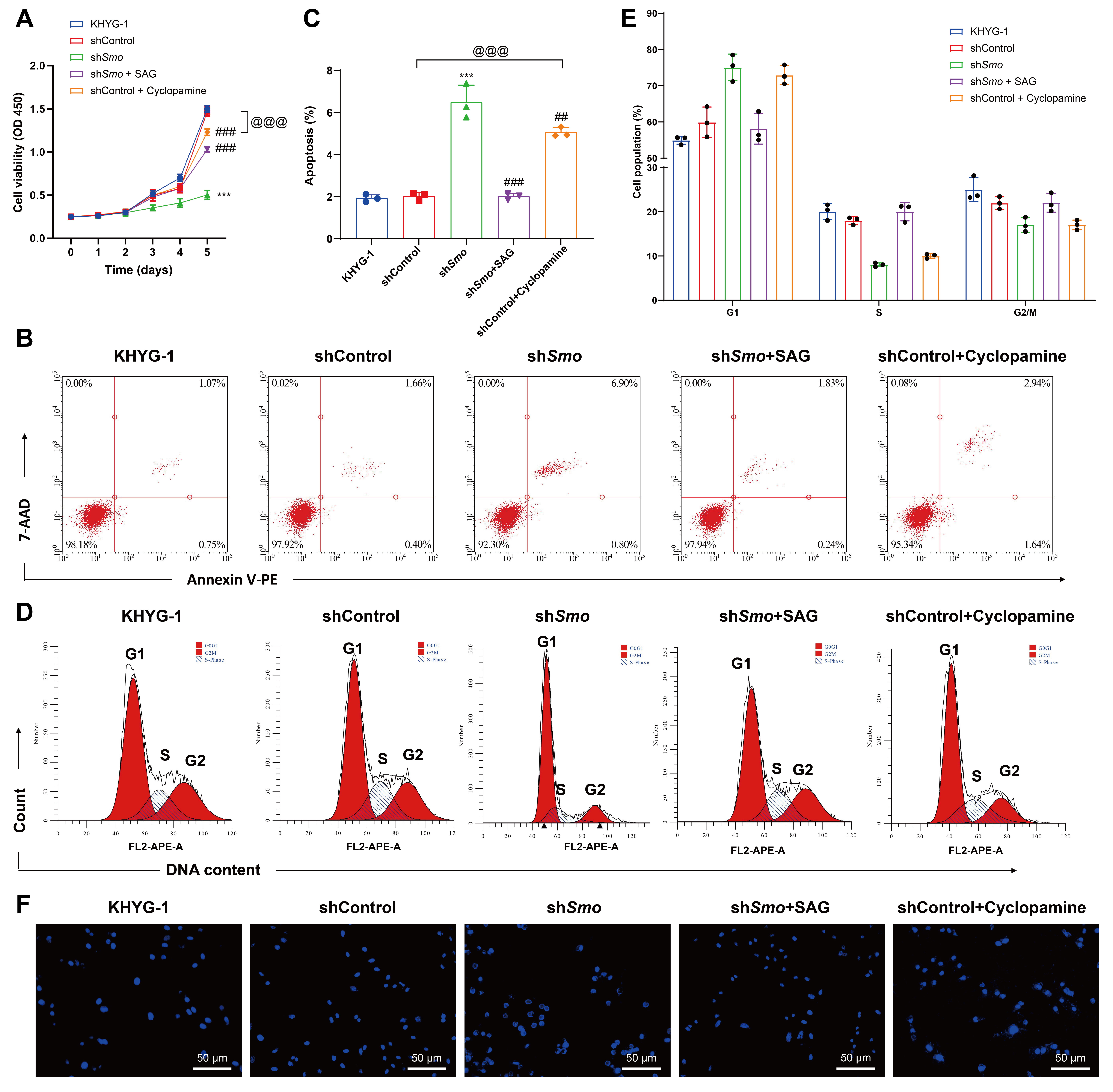

Supplement: Supplementary file 2 — Supplementary Material 2. [file 10020_2025_1341_MOESM2_ESM.jpg]

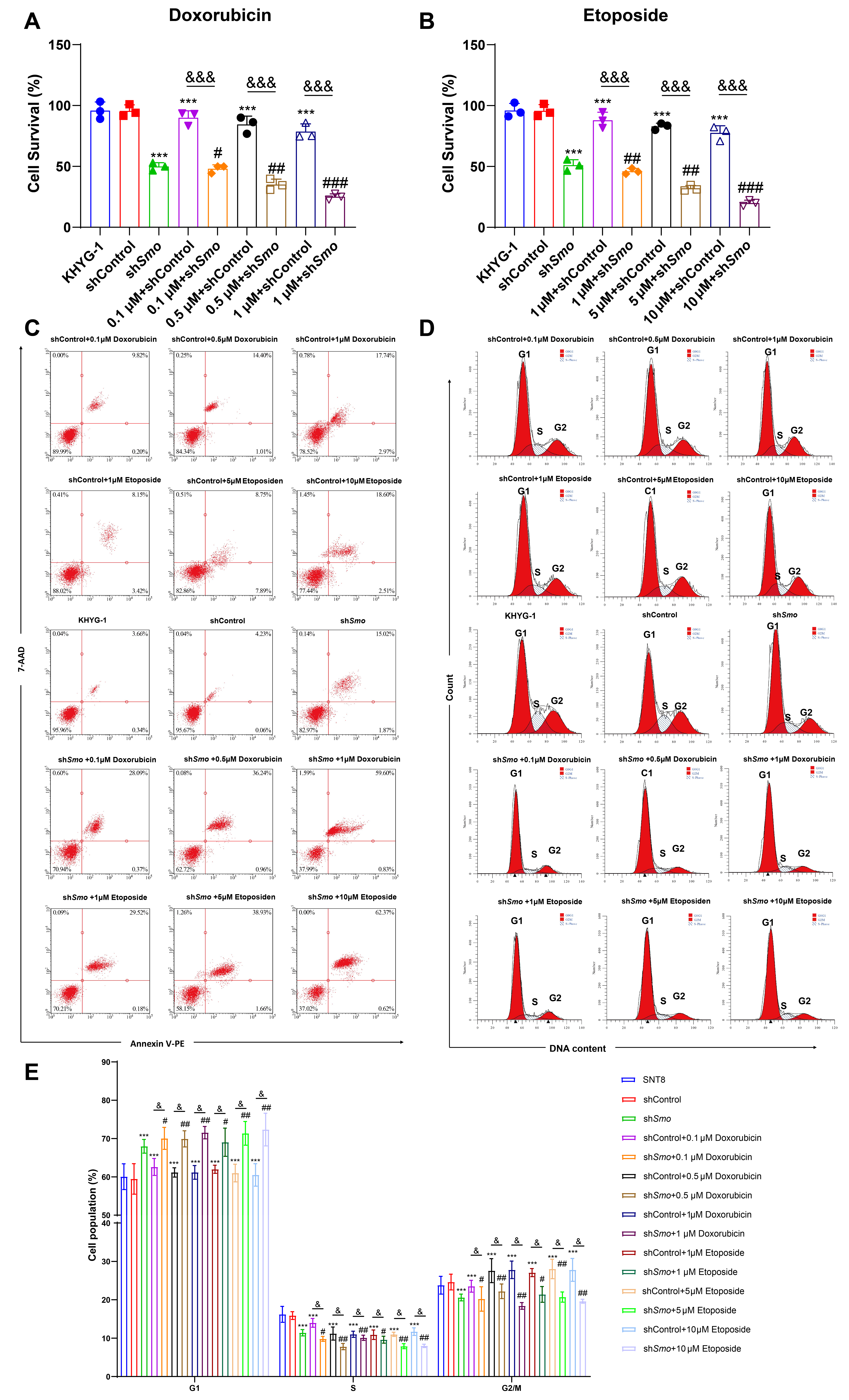

Supplement: Supplementary file 3 — Supplementary Material 3. [file 10020_2025_1341_MOESM3_ESM.jpg]

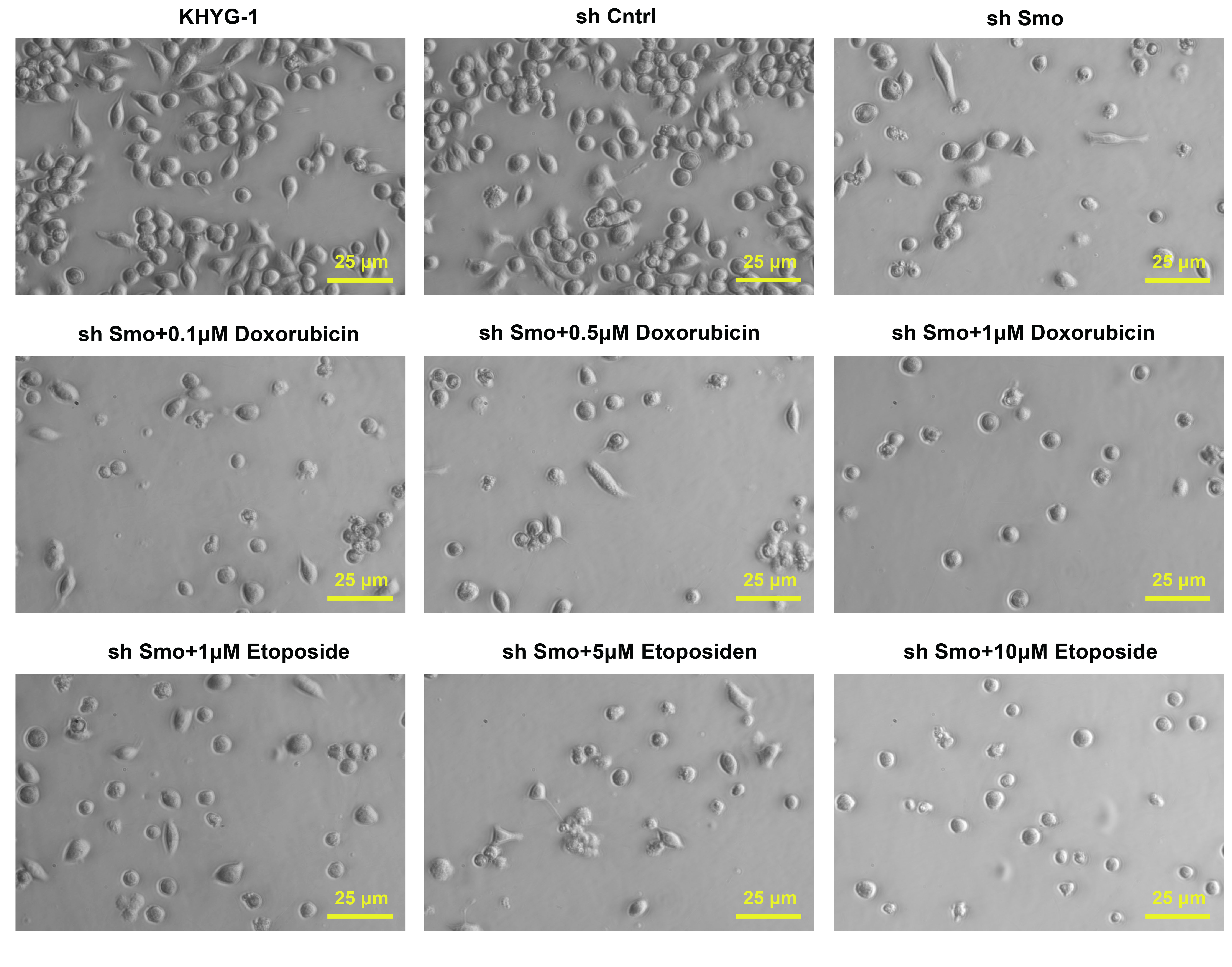

Supplement: Supplementary file 4 — Supplementary Material 4. [file 10020_2025_1341_MOESM4_ESM.jpg]

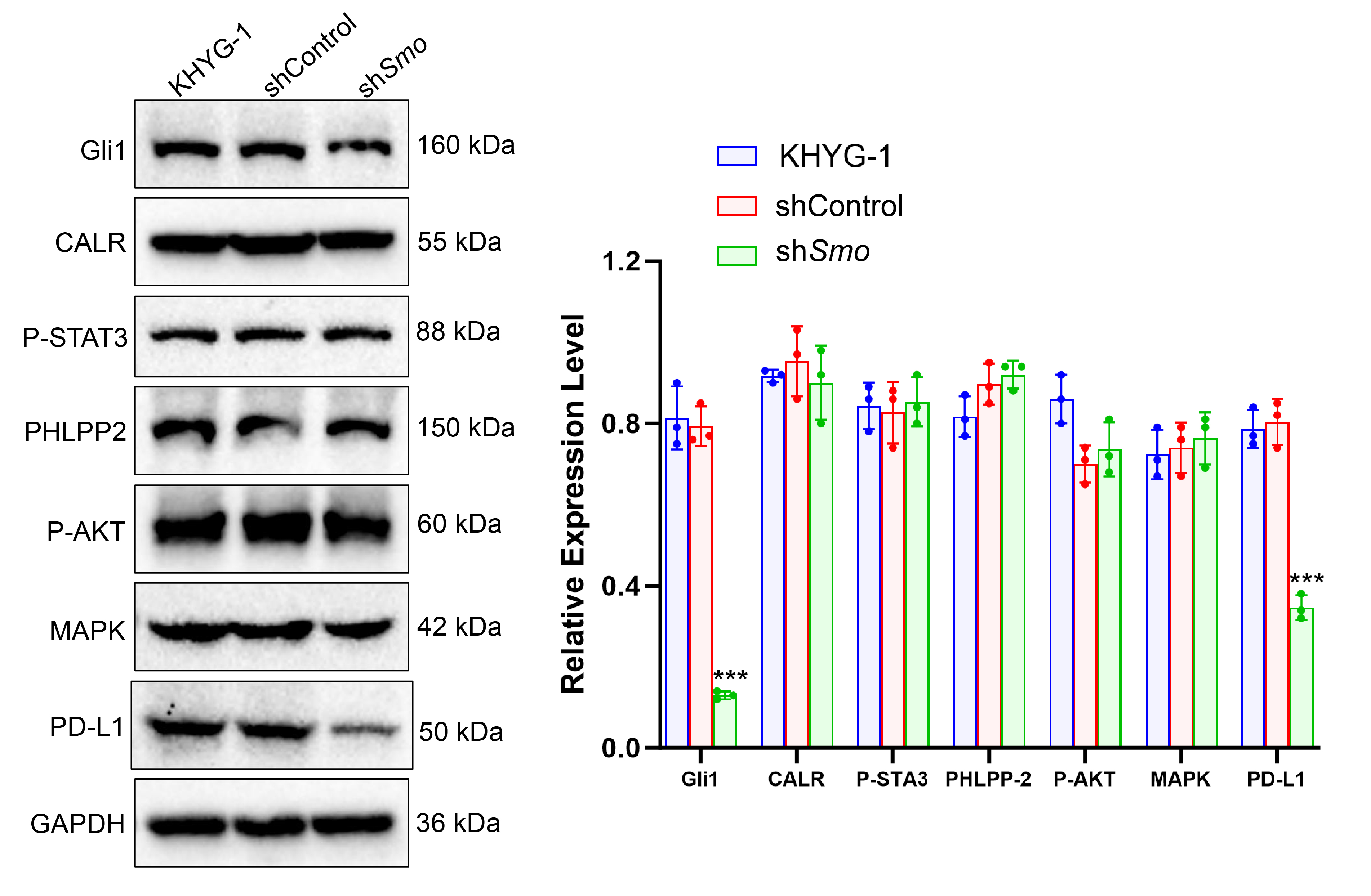

Supplement: Supplementary file 5 — Supplementary Material 5. [file 10020_2025_1341_MOESM5_ESM.jpg]

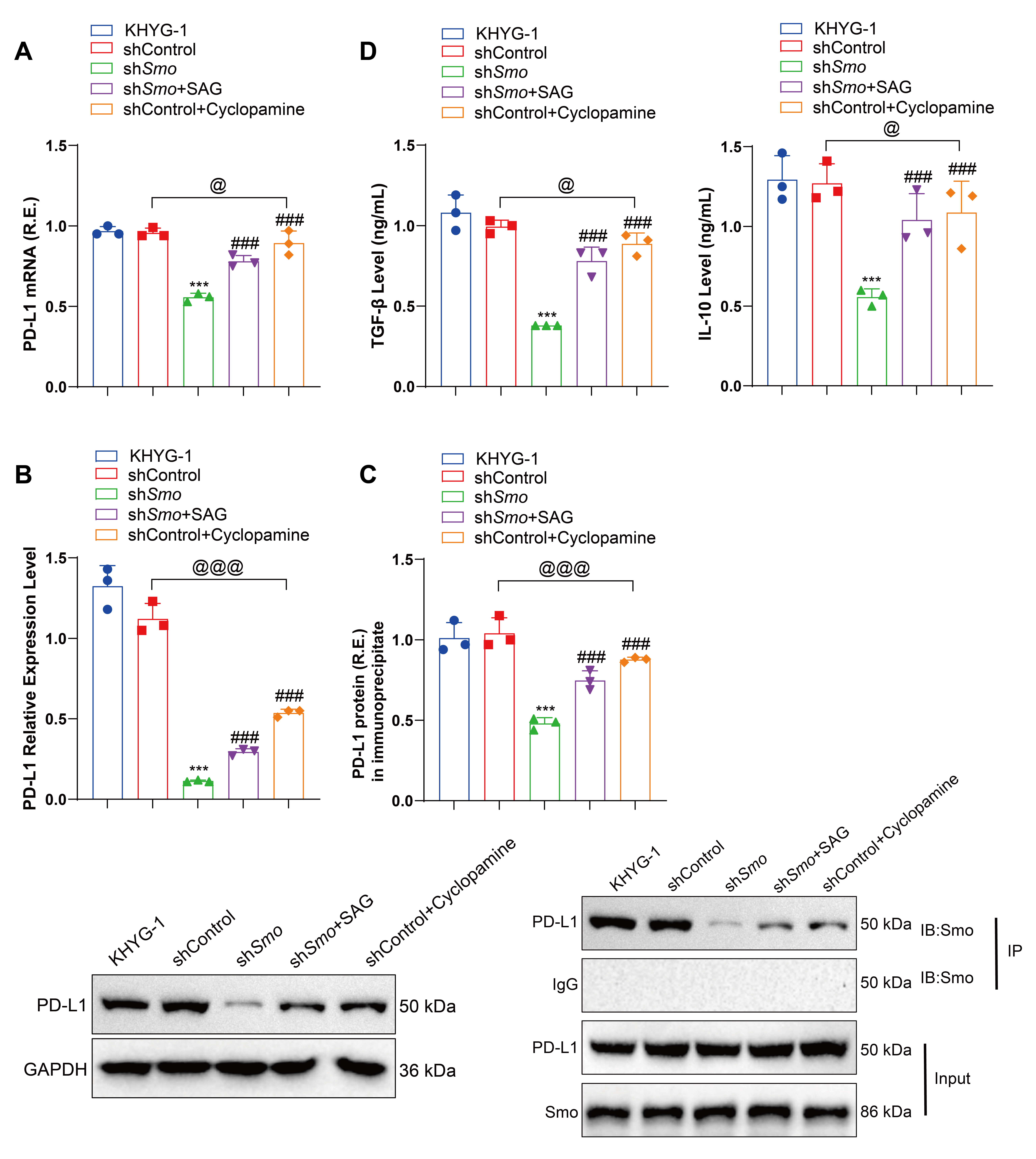

Supplement: Supplementary file 6 — Supplementary Material 6. [file 10020_2025_1341_MOESM6_ESM.jpg]
